# Electromyographic Evaluation of Muscle Activity in Patients Rehabilitated with Full Arch Fixed Implant-Supported Prostheses

**DOI:** 10.3390/medicina59020299

**Published:** 2023-02-06

**Authors:** Mihail Mostovei, Oleg Solomon, Nicolae Chele, Cosmin Sinescu, Virgil-Florin Duma, Andrei Mostovei

**Affiliations:** 1 Department of Prosthetic Dentistry, State University of Medicine and Pharmacy “Nicolae Testemițanu”, 165 Ştefan cel Mare şi Sfânt Ave., MD-2004 Chisinau, Moldova; 2 Department of Oro-Maxillo-Facial Surgery and Oral Implantology, State University of Medicine and Pharmacy “Nicolae Testemițanu”, 165 Ştefan cel Mare şi Sfânt Ave., MD-2004 Chisinau, Moldova; 3 Research Center in Dental Medicine Using Conventional and Alternative Technologies, School of Dental Medicine, “Victor Babes” University of Medicine and Pharmacy of Timisoara, 9 Revolutiei 1989 Ave., 300070 Timisoara, Romania; 4 3OM Optomechatronics Group, Faculty of Engineering, *Aurel Vlaicu* University of Arad, 2 Elena Dragoi Str., 310177 Arad, Romania; 5 Doctoral School, Polytechnic University of Timisoara, 1 Mihai Viteazu Ave., 300222 Timisoara, Romania

**Keywords:** dental medicine, full edentulism, implant rehabilitation, implant-supported prostheses, surface electromyography

## Abstract

*Background and Objectives*: Implant rehabilitation of complete edentulous arches has become more and more popular because of the increased access of the population to this type of treatment. Furthermore, the development of new rehabilitation procedures can be applied in most clinical cases, including in those with severe atrophy. Hence, this study aimed to assess the functional changes that can occur in the stomatognathic system after implant rehabilitation procedures. *Materials and Methods*: A total of 63 patients were accepted in the study. They were divided into a first control (dentate) group (CG) and a second study group (edentulous, SG). For the latter, 30 patients received 204 two-stage implants immediately loaded with provisional prostheses. Surface electromyography (EMG) was assessed at the time of prostheses fixation, while for some patients it was applied six months after the fixation of the fixed prostheses, as well. These supplemental investigated patients formed a third, follow-up study group (FSG). All assessments were performed during the processes of clenching and mastication. The obtained data of the two study groups, SG and FSG, were compared with those of the control group, CG. *Results*: No statistical differences were found in the electrical muscular activity between the study and control groups during both clenching and mastication (*p* > 0.05). In addition, there were no differences within the same study group, both initially and after 6 months. The only changes were noticed between static and dynamic values for the right masseter muscle in the follow-up group FSG (*p* = 0.008). Deviations of the overlapping coefficients were similar for all groups (*p* = 0.086): for CG, 20.5%, median 11.1 (min. 0, max. 104); for SG, 21.4%, median 12.2 (min. 0, max. 103); for FSG, 36.1%, median 26.9 (min. 0, max. 160). This revealed no neuromuscular adaption to the prostheses. *Conclusions*: Implant-prosthetic rehabilitation led to an EMG activity that was similar to that of dentate patients immediately after the placement of the fixed implant-supported prostheses. Moreover, the measured values did not change after six months of functioning for all evaluated parameters. This may point to an immediate restoration of the muscle contraction capacity, without the necessity of adaptation over time. The study serves as an argument for the application and reliability of the immediate fixed implant-supported prostheses from the perspective of muscle adaptation and functioning.

## 1. Introduction

Complete edentulism is an irreversible pathology that represents “the final marker of disease burden for oral health” [1]. Patients suffering from this pathology may have a decrease in mastication efficiency, esthetics, phonetics, and psychological status [2]. Several studies have proven the correlation between malnutrition and edentulism [3,4]. Thus, patients who tend to avoid hard unprocessed food such as fruits and vegetables have a decrease in the intake of vitamin C, magnesium, and calcium. Additionally, they have an increase in the intake of carbohydrates and lipids.

Implant-supported prostheses have become the treatment of choice, as well as the minimum standard of care in completely edentulous patients [5]. It has been demonstrated that the use of implants leads to better prostheses stability and improves the phonetics, mastication, and psychological status of fully edentulous patients [6,7]. It can be highlighted that one of the main objectives of the treatment is to restore the masticatory and muscle function in a way that would be equal or close to the one of dentate patients. Referring to this aspect, for a better understanding of the results of implant treatments, objective measurements of the patients’ functional parameters are necessary. In this respect, electromyography (EMG) is widely used in dentistry to diagnose different pathologies, including temporo-mandibular joint dysfunctions, dystonia, lesions of cranial nerves, muscle response after surgical procedures, as well as results of dental treatments [8,9,10,11]. For example, Ferrario et al. have shown that EMG can be considered as an important technique for the assessment of occlusal conditions [12]. Ximinis et al. utilized EMG to determine changes that occur during placement of fixed implant-supported prostheses with different occlusal schemes [13].

Complete implant rehabilitations provided in a short period of time can lead to changes in the activity of masticatory muscles and of other structures related to teeth, as well as in their functioning [14]. In this respect, EMG has been chosen as the main investigation method in our direction of research regarding the assessment of effects of implant-supported fixed prostheses on the patients’ functional parameters, with respect to both masticatory and muscle functions. Implant-supported overdentures have been proven to increase stability, comfort, and functioning in comparison to previously removable conventional prostheses. They also provide a higher value of EMG activity in comparison to conventional dentures [14,15]. Implant overdentures have provided better results when the number of implants was increased, thus leading to better stability of the prosthesis [16]. Thus, a literature review published by von der Gracht in 2016 has shown that EMG activity in implant overdentures and fixed implant-supported dentures is significantly higher than in conventional dentures. However, it is lower when compared to dentate subjects during chewing [17].

It is worth mentioning that the results provided in the literature are controversial and might be influenced by different factors that lead to an increased or decreased muscular activity compared to dentate subjects [14,18,19]. In consequence, this is an issue that must be studied, especially as the rapid development of implantology has led to an increase in complications related to implant treatments. They are classified as surgical complications (i.e., bone loss, implant loss, and peri-implant soft tissue complications), mechanical complications (i.e., screw loosening, chipping, and prosthesis fracture), as well as esthetic/phonetic complications [7,20]. One of the most common complications is the chipping of the veneering material [21], which in implant rehabilitation can be caused by the diminished sensory output from the absent periodontal ligaments [22].

Neuromuscular adaptation to the newly inserted prostheses can also play a role in the EMG activity, biting forces, and food perception; this role might increase over time [23,24]. However, there are no sufficient data in the literature concerning changes in EMG activity in the same group of rehabilitated patients with fixed prostheses over time (both in mastication and in maximum voluntary contraction (MVC)) compared to dentate subjects. In this respect, the fast development of biomaterials, technology, and techniques used in dentistry creates an increasing number of variables that can influence the tissues around implants, force distribution, adaptation, and muscle function [25,26].

Considering the above issues, the aim of this work was to assess the functional changes that can occur in muscles of mastication after implant rehabilitation with fixed screw retained prostheses immediately after fixation as well as in a short-term perspective of six months. Several research hypotheses were proposed to be tested through the present study:

- The first hypothesis was that the EMG activity would change over time due to the neuromuscular adaptation of the patients to newly inserted prostheses.
- The second hypothesis was that EMG activity in edentulous subjects is higher than in dentate ones due to the lack of periodontal mechano-receptors.
- The third hypothesis was that EMG activity in rehabilitated patients with fixed implant-supported prostheses is higher during mastication in comparison to the control (dentate) group.

## 2. Materials and Methods

The present study was approved by the Ethics Committee of the State University of Medicine and Pharmacy “Nicolae Testemitanu”, Chisinau, Moldova, Ethical Approval no. 43 from 16 March 2018. The study was designed as a prospective clinical control trial. The sample size was calculated using the following parameters: effect size dz = 0.5; α err prob = 0.05; power (1-β err prob) = 0.8; noncentrally parameter δ = 2.5854415; critical t = 1.7062592; Df = 25.7380304, with a total sample size of 28 subjects for each group.

A total of 63 patients were included in this study, examined, and for the study group, treated in two University dental clinics, from March 2018 till September 2021. They were divided into two main groups, as follows:

*The first, study group (SG)* consisted initially of 37 patients (28 women and 9 men), considered within a close age range of 59 ± 1.44 years. From this group, 7 patients were excluded because of prosthesis fractures with loss of fragments (1 case), refuse of mastication test (3 cases), or zero EMG signal (3 cases). The patients of this group were completely edentulous or partially edentulous, without the possibility to preserve the remaining teeth. The inclusion criteria were no absolute contraindications to implant placement, no allergy to polymethyl methacrylate, no general or local muscular disorders that might influence the acquired EMG results, and no pacemakers.

For the considered patients of this group, a total of 204 implants were inserted using the following systems: 104 Fast and Fixed concept Bredent Sky (Bredent GmbH, Senden, Germany), 65 Dentium Superline implants (Dentium, Cypress, CA, USA), and 35 implants with internal hex Alpha-Bio Tech (Alpha-Bio Tech Ltd., Tel Aviv, Israel). The implants were immediately loaded with implant-supported dentures with a minimum of 10 teeth per arch. They were manufactured from acryl reinforced with a cobalt chrome metal framework that was made using the selective laser melting technique. Extensions in the dentures were avoided in order to keep from overloading the newly inserted implants. All prostheses were inserted in the first 7 days after implant placement. This can be considered as an immediate loading according to the ITI consensus [27].

*The second, control group (CG)* consisted of 33 dentate subjects (21 women and 12 men), aged 43 to 67 years. Patients who presented more than 2 units of fixed prostheses on one arch or edentulous areas for more than one tooth were excluded from the study. Thus, only patients who had class I malocclusion were accepted in the study. Due to the fact that the age range of the control group was 54 ± 1.26 years, most of them (i.e., 25 patients) had different degrees of teeth attrition.

*A third, follow-up study group (FSG)* was formed in addition to the two groups above. It consisted of subjects from the first, SG group, who came for control six months after the implant loading.

**Figure 1:**
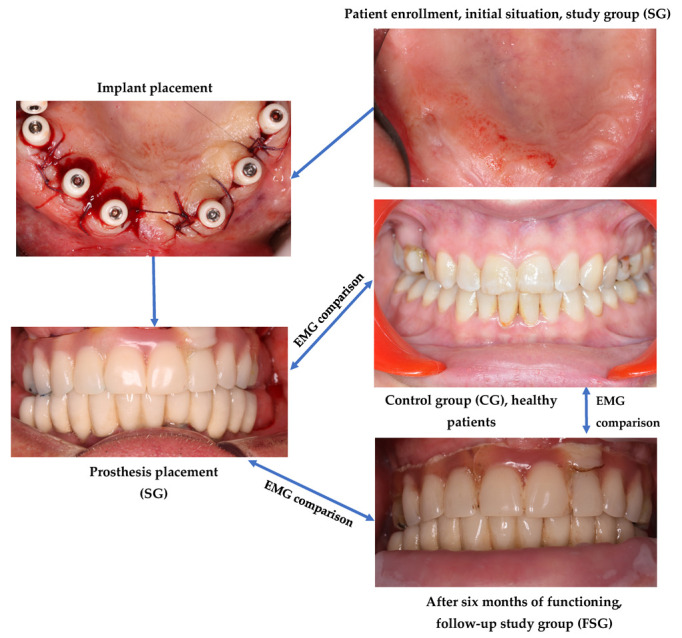
Treatment workflow of patient included in the study and directions of comparison between the groups.

All considered patients were subjected to clinical and paraclinical examinations in order to determine the available quantity of bone for implant placement. Additionally, a general health status check was completed. Patients from the SG received two-piece dental implants that were immediately loaded. EMG analysis of masticatory muscle function (i.e., masseter and temporal) in static regime and during chewing of 5 g of almonds were performed immediately after fixation, as well as (for FSG) after six months of functioning. The obtained statistical data were used to perform comparisons between groups. Figure 1 presents a scheme of this workflow that is detailed in the following.

Orthopantomographic X-rays were performed before the surgery, immediately after, as well as during the follow-up visit (i.e., after six months). The utilized radiography equipment was Orthopantomograph OP300 (Instrumentarium dental, PaloDEx Group Oy, Tuusula, Finland). Its main characteristics are resolution 100 µm, anode voltage from 57 to 90 kV, current intensity from 4 to 16 mA, exposure time from 8.6 to 16.1 s, and radiation dose from 47 to 131 µGycm^2^ [28,29].

In all the cases where there was a bone deficiency, Cone Beam Computed Tomography (CBCT) was used to assess anatomical conditions and landmarks. The same system as above was utilized, but with a resolution of 85 to 300 µm, 10 to 20 s exposure time, 2.34 to 12.5 s pulsed X-ray, and radiation dose from 193 to 385 µGycm^2^.

For the EMG data acquisition, a 4-channel Electromyograph was used, with concentric electrodes (ForEMG, Quatrotii, Italy). The technical characteristics of the equipment are input dynamics 18.75 mVPP, noise level reported at the input < 2 µV_RMS_, input impedance 500 MΩ, and frequency 2000 Hz. After a procedure of skin cleaning with 60% alcohol, the electrodes were placed on the masseter and temporal muscles of each subject.

**Figure 2:**
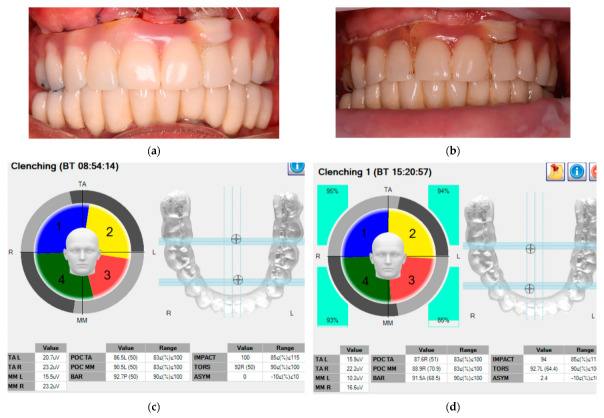
Evaluation of the EMG activity during maximum voluntary contraction (MVC) of newly inserted prosthesis: (**a**) clinical view after fixation; (**b**) clinical aspect after 6 months. The analyses in (**c**,**d**) represent graphic interpretations of the EMG activity for the situations presented in (**a**,**b**), respectively.

The data were collected twice: first, during the static maximum voluntary contraction and second during chewing of 5 g of almonds. In order to obtain an MVC, the patients were instructed to rest for 3 s, then clench their teeth for another period of 3 s, according to the recommendations made by Ferrario [12]. The patients had a rest between the data acquisition periods in order to avoid muscle fatigue. The same procedure was performed both during prostheses fixation and after six months. This specific follow-up period was selected according to the assumption that a neuromuscular adaptation occurs after six to twelve months, according to the literature [13,14]. The same measurements were performed again by the same prosthodontist, referring to the same prostheses, prior to their removal—as shown in the example in Figure 2.

Ten parameters were acquired throughout the study, as pointed out in Figure 2:

TAL—left temporalis muscle activity;

TAR—right temporalis muscle activity;

MML—left masseter muscle activity;

MMR—right masseter muscle activity;

PocTA/PocMM—percentage of the overlapping coefficient of temporalis/masseter, which indicates the overlapping during the functioning of these muscles;

Bar—barycenter, indicating the activity distribution between the masseters and temporalis muscles to anterior or posterior;

IMPACT—coefficient of muscle work intensity;

Tors—torsion coefficient of mandible during function that may appear due to a non-balanced occlusion;

Asym—inequality of right and left muscles during activity.

The first four parameters (i.e., TAL, TAR, MML, and MMR) represent the EMG activity of four masticatory muscles. They can be evaluated both as a mean value in a time span or as raw data. For our study, we chose to analyze the average values provided in the assessment period.

The other six parameters represent the interaction between the first four parameters. They provide insight on the muscular balance and the tooth contact distribution. These coefficients are expressed as deviations from the normal range of values. They have been considered in the present study because it is necessary to assess the correlation of the muscle’s activity during the fixation of the prostheses. Furthermore, they provide insight on the force distribution on a prosthesis and allow us to assess the further overall deviation of the patient from the normal range provided by the manufacturer.

The initial data were statistically analyzed using the Rstudio software (Posit, Boston, USA). The mean value with standard deviation (SD), the mean value with interquartile deviation (IQR), as well as the maximum and minimum values were evaluated for the considered continuous variables. The different distributions were assessed using the Shapiro–Wilk test. A comparative analysis was performed using non-parametric tests, depending on the correlation between the groups (i.e., variations of Wilcoxon tests) for the dependent and independent groups. The considered reference value (α) was 0.05. Data visualization was performed using the box plot variant combined with the jitter plot, or using the jitter plot combined with the violin plot.

## 3. Results

The general characteristics of the muscle activity, as well as the comparison between the different groups, are presented in Table 1. According to these data, there were no statistical differences between the groups regarding the muscular activity in MVC. It is also worth mentioning that the distribution of EMG activity in the examined muscles was rather high for all groups. This is in accordance with the work of von der Gracht [17].

### 3.1. Study Group (SG) vs. Control Group (CG)

**Figure 3:**
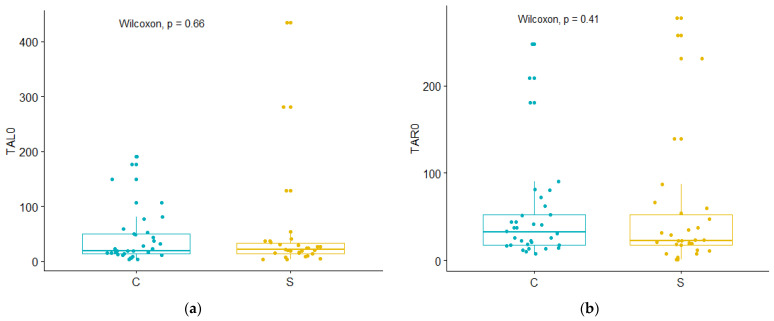

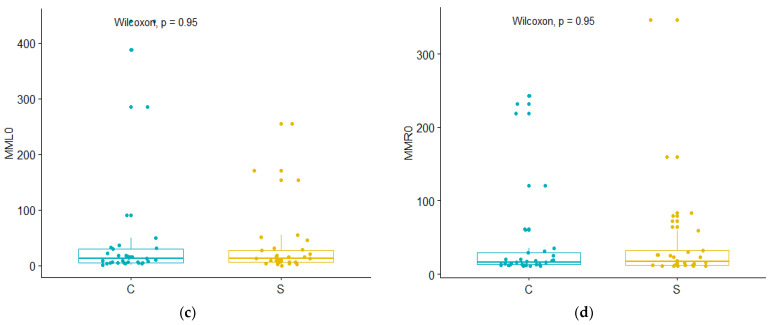
Comparative evaluation of muscle activity between the study group (S) and the control group (C): (**a**) TAL0—left temporalis muscle; (**b**) TAR0—right temporalis muscle; (**c**) MML0—left masseter muscle; (**d**) MMR0—right masseter muscle.

The data obtained in our study point to results similar to those previously described by Dellavia and by other authors [18], as presented in Figure 3. Thus, there were no statistical differences found between the study and control group (TAL0 for *p* = 0.66; TAR0 for *p* = 0.41; MML0 for *p* = 0.95; MMR0 for *p* = 0.95). This can indicate that fixed implant-supported restorations can immediately restore the complete muscle functions during MVC to levels comparable to dentate patients. The maximum registered levels during MVC were slightly higher in the study group and in the follow-up group. However, this had no impact on the statistical results. This aspect might be explained by the large distribution of values in the respective groups.

### 3.2. Study Group (SG) vs. Study Group after Six Months (FSG)

Comparative evaluations of the same patients after a period of time have the scope to determine if there were any changes in the muscular activity over time. This could indicate the degree of adaptation to the new dental prostheses. The influence of different dental materials is excluded because the same prostheses were evaluated for the same patients. Additionally, all provisional prostheses were made from the same materials, i.e., acrylic resin reinforced with a metallic framework.

According to the statistical analysis, the following values were obtained: **TAL** log_e_(V_Wilcoxon_) = 4.93, *p* = 0.148, r = −0.32, CI_95%_[−0.63, 0.08], n _pairs_ = 30; **TAR** log_e_(V_Wilcoxon_) = 5.17, *p* = 0.764, r = −0.07, CI_95%_[−0.45, 0.33], n _pairs_ = 30; **MML** log_e_(V_Wilcoxon_) = 5.37, *p* = 0.948, r = −0.02, CI_95%_[−0.40, 0.37], n _pairs_ = 30, and **MMR** log_e_(V_Wilcoxon_) = 5.56, *p* = 0.190, r = 0.29, CI_95%_[−0.12, 0.61], n _pairs_ = 30.

**Figure 4:**
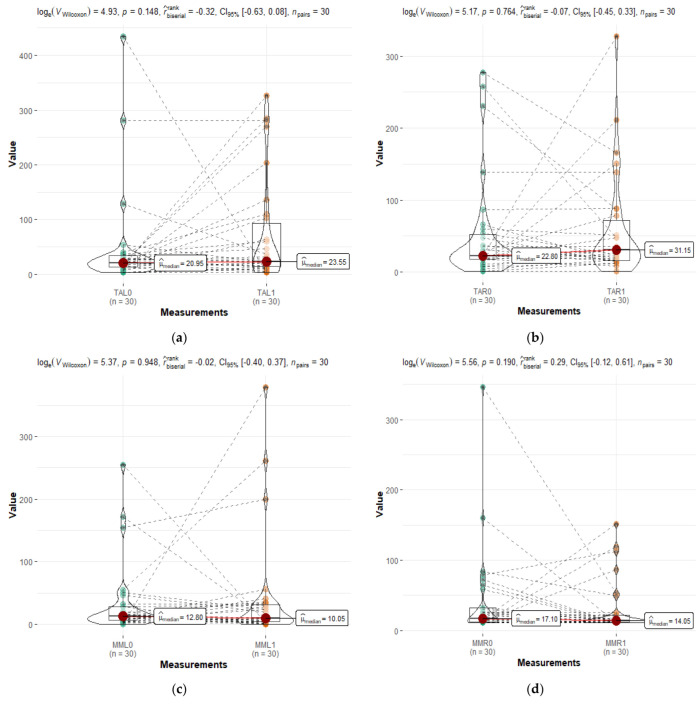
Comparative evaluation of the muscle activity between the study group (SG) and the follow-up group (FSG): (**a**) TAL—left temporalis muscle activity; (**b**) TAR—left temporalis muscle activity; (**c**) MML—left masseter muscle activity; (**d**) MMR—left masseter muscle activity. All parameters were considered initially (0) and after 6 months (1).

It can be seen from Figure 4 that there are some changes in time within the study group, but the median value shows almost no changes. The obtained *p* value was higher than 0.05 for all evaluated parameters. In comparison, in a study by Giannkopoulous, an adaptation to newly inserted prostheses after 3 months was shown [30]. However, there were no statistical differences observed between our groups, indicating that there were no changes in the muscular activity.

### 3.3. Study Group after Six Months (FSG) vs. Control Group (CG)

**Figure 5:**
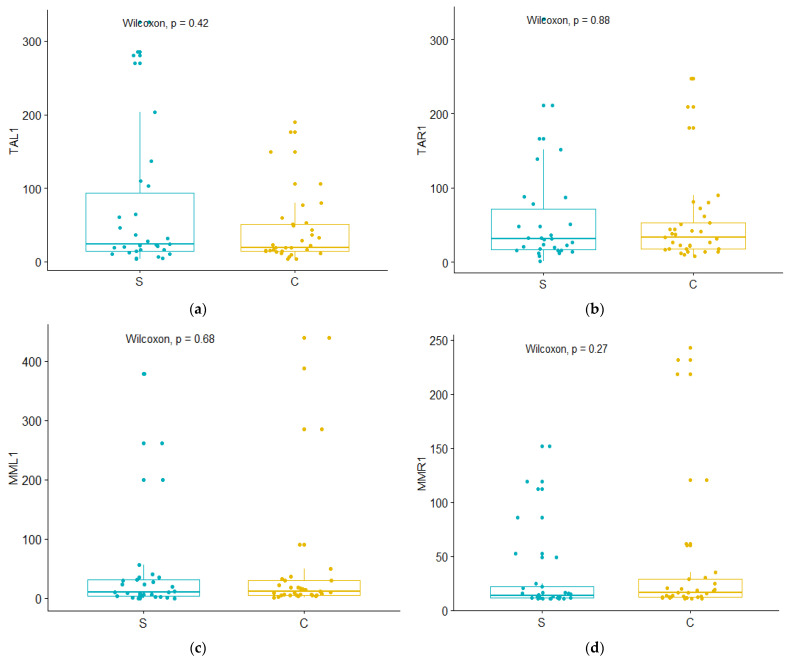
Comparative evaluation of the muscle activity between the study group after six months (S) and the control group (C): (**a**) TAL1—left temporalis muscle; (**b**) TAR1—right temporalis muscle; (**c**) MML1—left masseter muscle; (**d**) MMR1—right masseter muscle.

The comparison between the control group and the study group after six months of wearing prostheses showed no difference in the EMG activity of the patients wearing fixed prostheses on implants in comparison to dentate patients. This is demonstrated by the graphic presentations of the statistical analyses shown in Figure 5. In all four categories, the *p* value is higher than 0.05, indicating that there were no statistical differences between these groups (TAL1 for *p* = 042; TAR1 for *p* = 0.88; MML1 for *p* = 0.68; MMR1 for *p* = 0.27).

### 3.4. EMG Activity during Mastication

The analysis of the EMG activity during mastication can provide an insight into the muscle activity during dynamic movements. This activity could differ from the static regime because the patient is not always able to control the chewing forces. There are numerous studies that indicate different EMG activity during static and dynamic functioning of masticatory muscles [14,18]. This depends on several factors, including prostheses type, food type, and number of implants [31]. Some authors consider that the differences during mastication are produced by a lack of coordination between the muscle and the periodontium. This issue is absent in the case of implants [32]. A detailed description of the statistical analysis of the mastication is provided in Table 2.

It is worth mentioning that there were no statistical differences during mastication and MVC within the same group and between groups. The only difference was observed between the CG and FSG during chewing only in the right masseter muscle (*p* = 0.008331). We decided not to consider this as an important indicator because of the lack of other differences.

### 3.5. Deviation Coefficients

One of the issues that researchers have faced is the inability to compare the overlapping coefficients between different patients because such coefficients are the result of the interaction between subject-related variables, food texture, consistency, or weight [33]. This indicates if the treatment of a patient corresponds to the normal range given by the manufacturer for the six additional parameters considered in this study: PocTA, PocMM, Bar, IMPACT, Tors, and Asym. Their static and dynamic analysis is presented in Table 3. According to the obtained data, the patients were characterized by values that are close to the normal range. Still, a large distribution is noticed from the maximum and minimum acquired values.

It is possible to calculate how much each patient deviates from the normal range and then determine the mean deviation. The obtained parameter can indicate how much the values that characterize patients from the control group CG deviate from the given ranges and how the patients from the study group relate to the dentate ones. The range of each parameter provided by the manufacturer presented an acceptable normal deviation, as shown in Table 4. Therefore, we decided to calculate the percentage deviation of the specific value from the lower or the upper margin of the range, depending on the obtained result (i.e., if this result is above or below the specific range, respectively). In addition to the above-mentioned parameter, we calculated the deviation to the left or to the right, which is produced by the device software depending on which muscle group is dominant for a specific patient (i.e., left-right or front-back, respectively). A value of 0 (left and anterior) or a value of 1 (right and posterior) was provided for each parameter. Each of these parameters can be characterized only as left-right or anterior-posterior; therefore, it is impossible for the same parameter to have both a 0 and a 1 value. The obtained statistics are provided in Table 3 and Table 5.

Although deviations exist in different directions in all groups, including in the control one that had no dental procedure performed (or only a minimal one), the comparison demonstrated no statistical differences, where *p* > 0.005. Moreover, there were no statistical differences between the SG and FSG. This means that the initial deviation of the overlapping coefficients had the same direction even after six months of functioning. In our opinion, this indicates again the lack of muscular changes and adaptation over time to newly inserted prostheses.

The mean percentage deviation coefficients for the groups were as follows: for CG, 20.5%, median 11.1 (min. 0, max. 104); for SG, 21.4%, median 12.2 (min. 0, max. 103); for FSG, 36.1%, median 26.9 (min. 0, max. 160). For the latter one, we noticed an increase of 14.7% compared to the initial value; however, *p* = 0.086, which indicates a lack of statistical difference. In contrast to the first deviation parameter that indicates the direction of deviation, the second one (i.e., the mean percentage deviation) indicates how much the patient deviates from the normal range. In this case, it is worth noticing that the control group had no major dental procedures performed, and it still had a deviation of 20.5% from the normal range. This is comparable to the study group, which was characterized by 21.4%.

## 4. Discussion

Fixed implant prostheses have the role to restore the lost functions of dental arches as close as possible to dentate patients. The use of such fixed implant-supported prostheses has proven to be a better choice for patients with complete edentulism, as it provides better EMG activity and masticatory functions in comparison to the case of removable prostheses [7]. Other studies approached the impact of single- vs. two-implant retained overdentures on the muscular activity [34].

There are numerous works that indicate increased EMG activity in the case of removable implant-supported prostheses compared to conventional ones [35,36]. This is explained by some authors as a result of the increased stability of removable dentures because of the implanting–attaching systems [26]. Elsyad et al. showed in a recent study changes in the EMG activity related to the increased number of implants in the overdentures [37]. However, the overdentures with four implants used in that work corresponded more to the RP4 by Misch, which have full implant support without a mucosal component; biomechanically, such overdentures are more similar to fixed prostheses [38]. The fixed implant-supported dentures are more accepted today by patients due to the comfort and functioning they provide. However, different studies have pointed out different values of the EMG activity when using fixed implant-supported dentures, as compared to dentate subjects. This is in contrast to the goal of the treatment, which is to provide a rehabilitation as close as possible to natural dentition. A study by Jacobs demonstrated a higher EMG activity in patients with implant-supported prostheses compared to conventional dentures; however, dentate patients presented higher values of EMG activity compared to overdenture wearers and fixed ones [39]. However, it is worth mentioning that in that study, the dentate subjects were much younger than the edentulous ones. Additionally, all considered subjects were women. In contrast, other studies showed no differences between groups [17,18].

### 4.1. Electromyographic Activity in MVC

Within the limitations of this study, we were able to observe that the contracture capacity of masticatory muscles is immediately restored to the level of dentate patients. This is in contrast to other studies that mention a required time of several months for neuromuscular adaptation to the newly fixed prostheses [18,23]. However, to be exact, it is actually not possible to demonstrate that the muscular activity was truly restored in such patients because it is impossible to appreciate what the EMG parameters were when they had natural teeth.

The comparison of our study group (SG) immediately after prostheses fixation and after six months (i.e., in FSG) has shown no statistical differences in terms of muscular activity. In fact, this indicates that the muscle activity did not change in terms of contracture capacity. Similar values of the EMG activity were noticed in all three groups, even though the number of teeth was smaller in the study group. On the other hand, the obtained values had a high distribution in all the three groups. This latter conclusion can be found in the literature as well. Thus, values that vary between 58 and 320 µV for implant-supported prostheses and between 66 and 520 µV for dentate patients have been determined [17]. However, even though in some patients the muscular activity increased and in others it decreased over time, this proved to be insignificant for the mean values.

Even though the tooth types were different in the control group (CG, i.e., with natural teeth) vs. the study group (SG), there were no signs of muscular hyperactivity for the latter. Tanaka et al. mentioned in a recent research that full implant rehabilitation can lead to a lack of control over biting force [23]. Considering the lack of periodontal receptors in implants, the EMG values were not higher in our study group compared to the control group. This is contrary to the data obtained by the De Rossi [14,40], where there was higher EMG activity during clenching and posture. This might be explained by the specific implant types used in that work; they were zygoma implants, which in the end can provide different sensory outputs and biomechanics. In contrast, most of the prostheses utilized in the present study were manufactured according to the Fast and Fixed concept. These are closer to those in the study of Bersani et al., in which the Branemark protocol was used [14]. In the latter, lower values of EMG activity during MVC were observed in the study group compared to the control one. These results are similar to those obtained by Fereario et al., where dentate patients showed an increased EMG activity over implant-supported prostheses, both fixed and removable [19]. Moreover, both types of implant prostheses (i.e., removable and fixed) behaved similarly. This might be explained by the small number of subjects included in their study (i.e., 19 patients).

The overlapping coefficients have shown a symmetrical distribution in all the groups in our study, with the mean values within or close to the normal range (Table 3). These data are similar to those in Dellavia’s study, where three groups of patients were compared. The first group consisted of patients with All-on-4 implant denture in the mandible and upper removable; the second group consisted of patients with fixed implant dentures on both arches; the third group was the control dentate one [18]. In the research provided by Ferrario et al., there were also no differences in the overlapping coefficients between fixed implant prostheses wearers and healthy subjects [19]. By comparing the mean deviation coefficient, we can conclude that although control patients had a minimal dental treatment in their life, there was still no ideal matching into the normal range, which had values deviated by 20.5%. The majority of the rehabilitated patients included in the present study had 10 to 12 teeth on one or both arches. This might diminish the number of teeth contact and muscle activity. Therefore, there might be an effect on the overlapping coefficients because of the lack of enough back teeth. However, according to the statistical data shown in Table 3, this did not happen—both the overlapping and the deviation coefficients were similar in all three groups. This coefficient was used in our study to assess if normal ranges of the overlapping coefficients provided by the manufacturer can be considered reliable for the considered population sample. In this context, adjustments could be made to the ranges in the EMG values if a larger number of healthy populations could be enrolled in the study. An issue that we concluded is that it is impossible to compare the results obtained with the mean deviation coefficient with literature data due to a lack of similar studies and different study samples of different ages and ethnicity.

### 4.2. Dynamic Analysis

It is worth mentioning that the EMG values did not increase even during mastication within the same group. Despite the idea that clenching would produce more muscle activity than chewing, there were no statistical differences in the present study between static and dynamic conditions in the data obtained within each group. This might be explained by the type of food that was utilized. Our patients used only almonds, which is a hard food and requires more force for chewing. Perhaps if the selected food was softer, the muscle activity would have decreased [14,18].

The muscle activity did not differ between groups as well. Thus, the data obtained during mastication were similar in the study and in the control group. Dellavia et al. used a different method of comparison for the muscular activity during chewing. They calculated the standardized global impact for each group included in their study, with tests on alternating one-side chewing [18]. As a result, they observed no statistical differences between healthy subjects and patients with All-on-4 prostheses. In our study, we did not use chewing tests for right and left sides separately. Instead, we decided to maximize the normal conditions of patients by asking them to chew on both sides until the deglutition reflex appeared. This was also stated by Ferarrio et al., who pointed out that the normal, bilateral chewing test would be better for implant rehabilitation to assess the force distribution from the occlusal surface, as well as the neuromuscular adaptation with EMG records [33]. De Rossi et al. obtained a slightly different result, with an increase in muscle activity for patients rehabilitated with zygoma implants [14]. The review published by von de Gracht showed higher EMG activity during chewing [17]. In our study, the overlapping coefficients were deviating from the normal range during mastication in all groups (Table 3), but this is a logical result considering the mastication pattern and the uneven contact with the bolus during mastication. We must highlight that in the present work, there was no aim to create an even pattern of mastication on both sides; patients were incentivized to chew automatically.

This study is part of a broader direction of research that included a previously published paper regarding the evaluation of masticatory efficiency [41]. In this respect, this previous study on the same patients showed statistical differences in the evaluated parameters between the control and study groups regarding chewing efficiency. It demonstrated a decrease in the masticatory efficiency of patients with fixed implant-supported dentures. Such patients had longer chewing time before deglutition, worse grinding capacity, and more chewing strokes than dentate subjects. This is in correlation with Trulsson Mats’ results, which concluded that patients with a lack of periodontal mechanoreceptors lack the “level, direction and point of attack of forces to hold and manipulate food between the teeth” [32]. This might explain the lack of sense during mastication: dentate patients can feel the food bolus and gently and efficiently move it to different teeth, thus providing a better mastication with the same EMG activity.

Limitations of the present study include the limited number of patients considered in each group. However, this number allowed for an appropriate statistical analysis of results. Future work is planned with larger study groups. Another limitation refers to the different number of teeth in the considered prostheses. This aspect is difficult to overcome in a clinical study, which includes real patients with specific medical needs. We envisage that future computational studies can add further information regarding the functioning of the periodontal system. Both analytical [42] and Finite Element Analysis (FEA) studies would be useful in this respect. This would be a valuable line of research, complementary to the clinical one. Other limitations of the present study may refer to the fact that the acquired data from muscles are impacted not only by occlusal contacts; they depend on other factors as well. Using the digital technology of direct tooth contact distribution, which is commercially available, would allow combining direct visualization of teeth contact with force distribution and muscle response.

## 5. Conclusions

The present study demonstrated that provisional implant-supported prostheses are capable of restoring the EMG activity immediately after prostheses fixation and that they do not require an adaptation period. The restored activity remained stable over a period that exceeded six months. The data acquired with EMG proved that provisionally fixed implant prostheses provided muscular activity that is similar to the dentate patients despite the lack of sufficient teeth. The deviations of overlapping coefficients from normal ranges are similar in all groups, including dentate one. They remained unchanged after six months because prostheses did not change over this period of time. Mastication has shown similar muscular activity when compared to the static condition. However, the overlapping coefficients in mastication deviated more from the normal range compared to MVC. Mean deviation coefficients have shown that even healthy patients in MVC do not perfectly match the provided normal range for Poc%, BAR, Impact, and Tors. The mean deviation coefficient was not previously mentioned in the literature. It can be used to provide a direct comparison of EMG parameters between groups or individuals despite the presence of some highly dissipated values referring to a specific parameter. Healthy subjects did not perfectly match in the normal ranges provided by the manufacturer. This might be due to some registration issues or to anatomical features of the specific population.

The first hypothesis was that muscles of mastication suffer changes in their activity over time due to neuromuscular adaptation. This hypothesis was rejected, because there were no statistical differences in the muscular activity immediately after prostheses fixation compared to the situation after six months of functioning.

The second hypothesis was rejected as well, as there were no differences in the EMG muscle activity between healthy subjects and patients with fixed provisional implant prostheses, despite the lack of periodontal ligaments and movement control in the latter. Muscles immediately developed equal parameters after prostheses fixation, similar to dentate subjects.

The third hypothesis referred to the fact that during mastication, the muscle activity is different from the static one due to the lack of maximum intercuspation during mastication. This hypothesis was rejected as well, as patients had similar muscular activity both during mastication and clenching. This can be due to the food type utilized in this study, which requires more force to be chewed.

Future studies are planned regarding permanent dentures for the same patients in order to exclude the variable related to the number of teeth, and in order to assess their effect on both EMG activity and mastication. The change of prostheses materials can further impact the acquired data, as well.

## Figures and Tables

**Table 1:** Descriptive statistics of the EMG activity in MCV for the control group (CG), the study group (SG), and the study group after the six-month follow-up period (FSG).

		CG (N = 33)	SG (N = 30)	FSG (N = 30)	Wilcoxon Test (CG vs. SG)	Wilcoxon Test (CG vs. FSG)	Wilcoxon Paired Test (SG vs. FSG)
**TAL** (µV)	Mean (SD)	42.0 (48.5)	46.9 (89.8)	73.6 (97.6)	W = 527, *p* = 0.6646	W = 554, *p* = 0.4207	V = 267, *p* = 0.1482
	Median (IQR)	18.8 (36.3)	21.0 (20.3)	23.6 (79.1)			
	[Min., Max.]	[3.80, 190]	[3.80, 434]	[3.80, 326]			
	Shapiro-Wilk normality test	W = 0.72061, *p* = 1.361 × 10^−6^	W = 0.72061, *p* = 1.361 × 10^−6^	W = 0.6981, *p* = 1.464 × 10^−6^			
**TAR** (µV)	Mean (SD)	51.4 (56.8)	53.5 (74.4)	59.4 (72.9)	W = 556, *p* = 0.4051	W = 483.5, *p* = 0.8797	V = 202, *p* = 0.7639
	Median (IQR)	32.9 (35.2)	22.8 (34.6)	31.2 (55.2)			
	[Min., Max.]	[7.90, 248]	[1.30, 278]	[1.30, 328]			
	Shapiro-Wilk normality test	W = 0.67751, *p* = 3.031 × 10^−7^	W = 0.67751, *p* = 3.031 × 10^−7^	W = 0.69981, *p* = 1.551 × 10^−6^			
**MML** (µV)	Mean (SD)	48.7 (107)	33.8 (57.7)	41.7 (85.4)	W = 490, *p* = 0.9506	W = 464.5, *p* = 0.6797	V = 221, *p* = 0.9483
	Median (IQR)	12.3 (24.8)	12.8 (21.2)	10.1 (27.6)			
	[Min., Max.]	[1.50, 439]	[0.200, 255]	[0.300, 379]			
	Shapiro-Wilk normality test	W = 0.45479, *p* = 5.957 × 10^−10^	W = 0.45479, *p* = 5.957 × 10^−10^	W = 0.50205, *p* = 5.634 × 10^−9^			
**MMR** (µV)	Mean (SD)	42.1 (64.4)	41.9 (66.1)	30.5 (37.1)	W = 500.5, *p* = 0.945	W = 415, *p* = 0.2734	V = 145, *p* = 0.1904
	Median (IQR)	16.3 (16.7)	17.1 (20.3)	14.1 (10.1)			
	[Min., Max.]	[11.4, 243]	[11.4, 346]	[11.3, 152]			
	Shapiro-Wilk normality test	W = 0.51507, *p* = 2.646 × 10^−9^	W = 0.51507, *p* = 2.646 × 10^−9^	W = 0.5765, *p* = 3.816 × 10^−8^			

Notations: TAL—left temporalis, TAR—right temporalis, MML—left masseter, MMR—right masseter, Mean (SD—standard deviation), Median (IQR—interquartile deviation), Min.—minimum value, Max.—maximum value, df—degrees of freedom.

**Table 2:** Descriptive statistics of the EMG activity during mastication for the control group (CG), the study group (SG), and the study group after the six-month follow-up period (FSG), as well as their comparison with MVC.

		CGch (N = 33)	SGch (N = 30)	FSGch (N = 30)	CG vs. CGch	SG vs. SGch	FSG vs. FSGch	SGch vs. FSGch	CGch vs. SGch and FSGch
**TAL** (µV)	Mean (SD)	41.4 (60.9)	46.7 (73.1)	74.9 (106)	**Wilcoxon paired test, V (p)**				**Wilcoxon test**
	Median (IQR)	25.9 (18.1)	20.3 (13.5)	21.4 (72.6)					
	[Min, Max]	[3.80, 301]	[3.80, 291]	[3.80, 442]	V = 263, *p* = 0.9925	V = 187, *p* = 0.9713	V = 249, *p* = 0.5027	V = 137, *p* = 0.1358	W = 534.5, *p* = 0.5914; W = 463, *p* = 0.6646
	Shapiro-Wilk normality test	W = 0.52469, *p* = 3.395 × 10^−9^	W = 0.54318, *p* = 1.581 × 10^−8^	W = 0.67237, *p* = 6.334 × 10^−7^					
**TAR** (µV)	Mean (SD)	49.0 (59.1)	66.6 (88.7)	68.5 (109)	V = 221, *p* = 0.4268	V = 291, *p* = 0.1144	V = 232, *p* = 1.000	V = 264.5, *p* = 0.517	W = 516, *p* = 0.7778; W = 547.5, *p* = 0.4742
	Median (IQR)	27.4 (26.1)	27.3 (50.2)	24.7 (21.7)					
	[Min, Max]	[5.60, 255]	[1.90, 305]	[2.90, 421]					
	Shapiro-Wilk normality test	W = 0.60284, *p* = 2.94 × 10^−8^	W = 0.68422, *p* = 9.27 × 10^−7^	W = 0.57884, *p* = 4.067 × 10^−8^					
**MML** (µV)	Mean (SD)	41.7 (96.0)	58.6 (85.3)	53.3 (111)	V = 186, *p* = 0.1473	V = 271, *p* = 0.1243	V = 228, *p* = 0.9344	V = 247, *p* = 0.5306	W = 394, *p* = 0.1666; W = 496, *p* = 0.9945
	Median (IQR)	10.1 (11.9)	14.6 (64.7)	10.5 (17.2)					
	[Min, Max]	[2.60, 439]	[0.600, 321]	[1.70, 458]					
	Shapiro-Wilk normality test	W = 0.42774, *p* = 3.163 × 10^−10^	W = 0.69698, *p* = 1.41 × 10^−6^	W = 0.50761, *p* = 6.454 × 10^−9^					
**MMR** (µV)	Mean (SD)	51.0 (89.3)	47.7 (82.1)	49.6 (78.7)	V = 293, *p* = 0.594	V = 270.5, *p* = 0.1271	V = 340, *p* = 0.008331	V = 211, *p* = 0.8644	W = 509, *p* = 0.8526; W = 552.5, *p* = 0.4327
	Median (IQR)	16.4 (16.9)	16.1 (15.2)	15.3 (15.0)					
	[Min, Max]	[11.4, 401]	[11.5, 397]	[11.4, 303]					
	Shapiro-Wilk normality test	W = 0.48789, *p* = 1.332 × 10^−9^	W = 0.49244, *p* = 4.462 × 10^−9^	W = 0.53966, *p* = 1.444 × 10^−8^					

Notations: TAL—left temporalis, TAR—right temporalis, MML—left masseter, MMR—right masseter, -ch—indicates the mastication in the respective muscle, Mean (SD—standard deviation), Median (IQR—interquartile deviation), Min.—minimum value, Max.—maximum value, df—degrees of freedom.

**Table 3:** Comparative static and dynamic analysis of the overlapping coefficients, Bar, Impact, Asym, and Tors for the three considered groups, showing their respective deviation percentages.

		CG (N = 33)	SG (N = 30)	FSG (N = 30)	CGch (N = 33)	SGch (N = 30)	FSGch (N = 30)
**POCTA**	Mean (SD)	74.7 (17.2)	74.9 (17.6)	67.0 (17.8)	62.3 (14.1)	63.1 (21.5)	56.9 (16.2)
	Median (IQR)	81.0 (18.9)	82.9 (20.7)	70.1 (22.3)	66.4 (22.2)	71.6 (29.7)	59.1 (19.6)
	[Min, Max]	[29.0, 90.9]	[29.8, 95.1]	[26.8, 88.8]	[34.8, 83.3]	[15.3, 89.6]	[16.8, 86.8]
**CD_POCTA%**	Mean (SD)	12.5 (18.8)	12.1 (19.4)	20.2 (20.5)	25.0 (17.0)	24.5 (25.4)	29.0 (20.0)
	Median (IQR)	2.41 (17.5)	0.0602 (20.2)	15.6 (26.8)	20.0 (26.7)	13.7 (35.8)	26.1 (28.0)
	[Min, Max]	[0, 65.1]	[0, 64.1]	[0, 67.7]	[0, 58.1]	[0, 81.6]	[0, 79.8]
**POCMM**	Mean (SD)	73.6 (17.2)	63.5 (24.5)	58.0 (25.9)	52.3 (15.5)	51.7 (21.5)	41.1 (23.2)
	Median (IQR)	76.2 (18.1)	68.4 (26.0)	58.1 (39.3)	52.7 (14.7)	54.6 (26.3)	40.8 (28.9)
	[Min, Max]	[14.5, 98.7]	[10.5, 90.5]	[7.20, 97.8]	[10.4, 85.1]	[8.30, 90.2]	[1.70, 94.9]
**CD_POCMM%**	Mean (SD)	13.8 (18.3)	21.9 (25.9)	31.7 (29.2)	37.0 (18.5)	38.1 (25.2)	50.1 (27.8)
	Median (IQR)	8.19 (18.9)	14.8 (30.5)	30.1 (47.3)	36.5 (17.7)	34.3 (31.7)	50.8 (36.1)
	[Min, Max]	[0, 82.5]	[0, 79.4]	[0, 91.3]	[0, 87.5]	[0, 90.0]	[0, 98.0]
**BAR**	Mean (SD)	76.4 (14.7)	73.6 (16.2)	72.6 (15.1)	71.4 (11.1)	65.3 (19.8)	59.1 (19.9)
	Median (IQR)	80.1 (21.4)	79.7 (23.5)	77.3 (19.2)	72.4 (16.8)	70.8 (24.2)	64.6 (25.9)
	[Min, Max]	[23.4, 93.1]	[39.8, 92.7]	[34.3, 91.5]	[48.5, 90.1]	[14.3, 91.2]	[8.40, 84.3]
**CD_BAR%**	Mean (SD)	15.4 (16.0)	18.4 (17.7)	19.3 (16.7)	20.7 (12.3)	27.5 (22.0)	34.3 (22.2)
	Median (IQR)	11.0 (23.8)	11.5 (26.1)	14.2 (21.3)	19.6 (18.7)	21.3 (26.9)	28.3 (28.8)
	[Min, Max]	[0, 74.0]	[0, 55.8]	[0, 61.9]	[0, 46.1]	[0, 84.1]	[6.33, 90.7]
**IMPACT0**	Mean (SD)	95.0 (28.2)	105 (22.8)	105 (63.9)	94.2 (24.8)	168 (177)	160 (149)
	Median (IQR)	95.0 (31.0)	100 (30.0)	97.5 (28.3)	91.0 (33.0)	103 (33.5)	117 (51.0)
	[Min, Max]	[0, 150]	[66.0, 164]	[0, 399]	[46.0, 146]	[55.0, 824]	[29.0, 724]
**CD_IMPACT0%**	Mean (SD)	9.87 (19.5)	6.46 (10.4)	16.9 (46.3)	9.30 (12.7)	60.9 (147)	57.6 (116)
	Median (IQR)	0 (11.3)	0.865 (7.74)	0 (10.2)	3.40 (16.0)	2.60 (14.9)	12.2 (46.7)
	[Min, Max]	[0, 100]	[0, 42.0]	[0, 246]	[0, 45.9]	[0, 616]	[0, 530]
**TORS**	Mean (SD)	80.5 (12.4)	76.1 (16.2)	72.8 (13.9)	69.3 (10.9)	68.7 (19.3)	55.7 (18.4)
	Median (IQR)	83.5 (15.8)	82.9 (24.8)	72.1 (15.9)	71.5 (11.4)	73.5 (25.2)	56.5 (36.6)
	[Min, Max]	[40.2, 93.9]	[40.7, 92.6]	[40.6, 92.7]	[37.6, 86.5]	[19.1, 89.1]	[27.2, 80.8]
**CD_TORS0 %**	Mean (SD)	11.2 (13.1)	15.8 (17.6)	19.3 (15.3)	23.0 (12.1)	23.6 (21.5)	36.3 (21.4)
	Median (IQR)	7.22 (17.3)	7.94 (27.5)	19.9 (17.7)	20.6 (12.7)	18.4 (27.9)	35.5 (41.1)
	[Min, Max]	[0, 55.3]	[0, 54.8]	[0, 54.9]	[3.89, 58.2]	[1.00, 78.8]	[0, 69.8]
**ASYM**	Mean (SD)	12.7 (13.5)	12.0 (12.4)	19.2 (16.3)	16.7 (16.1)	25.0 (24.8)	29.1 (23.6)
	Median (IQR)	6.60 (11.3)	8.30 (13.7)	17.2 (16.8)	9.30 (16.3)	12.8 (22.8)	25.4 (35.4)
	[Min, Max]	[0, 56.4]	[4.00, 53.8]	[0.300, 68.4]	[0.300, 54.3]	[0.400, 83.0]	[0.400, 80.1]
**CD_Asym %**	Mean (SD)	60.0 (114)	53.9 (99.5)	109 (147)	92.8 (141)	166 (235)	207 (219)
	Median (IQR)	0 (58.0)	0 (72.0)	71.5 (149)	0 (112)	28.0 (228)	154 (344)
	[Min, Max]	[0, 464]	[0, 438]	[0, 584]	[0, 443]	[0, 730]	[0, 701]

Notations: POCTA/MM—percentage overlapping coefficients, BAR—barycenter coefficient, TORS—torsion coefficient, IMPACT—index of muscle work intensity, Asym—asymmetry index, CD_%—deviation in percentage of respective coefficient, -ch—indicates the mastication in the respective group, Mean (SD—standard deviation), Median (IQR—interquartile deviation), Min.—minimum value, Max.—maximum value, df—degrees of freedom.

**Table 4:** Overlapping coefficient and the normal range provided by the manufacturer.

	Value		Value	Range		Value	Range
**TAL** (µV)	434.2	**POC TA**	80.6 L (98.6)	83 < (%) < 100	**IMPACT**	125	83 < (%) < 100
**TAR** (µV)	257.9	**POC MM**	80.7 R (77)	83 < (%) < 100	**TORS**	83.2 L (95.6)	90 < (%) < 100
**MM L**	10.4	**BAR**	80.7 A (93.5)	90 < (%) < 100	**ASYM**	−6.7	−10 < (%) < −10
**MM R**	11.4						

**Table 5:** Statistical analysis of the deviation for the overlapping coefficients for the three considered groups: CG, SG, and FSG.

	CG		SG						
Characteristic	n/N (%)	95% CI	n/N (%)	95% CI	n/N (%)	95% CI	Fisher’s test	McNemar’s test	Characteristic
**L0/R1_POCTA**							*p*-value = 0.3173, OR = 0.58 (95% CI 0.19, 1.73)	*p*-value = 0.6164, OR = 0.75 (95% CI 0.24, 2.27)	ꭓ^2^ = 0.071429, df = 1, *p*-value = 0.7893
**0**	13/33 (39)	23, 58	16/30 (53)	35, 71	14/30 (47)	29, 65			
**1**	20/33 (61)	42, 77	14/30 (47)	29, 65	16/30 (53)	35, 71			
**L0/R1_POCTAch**							*p*-value = 0.8014, OR = 0.82 (95% CI 0.27, 2.45)	*p*-value = 0.8025, OR = 1.21 (95% CI 0.40, 3.67)	ꭓ^2^ = 0.30769, df = 1, *p*-value = 0.5791
**0**	17/33 (52)	34, 69	17/30 (57)	38, 74	14/30 (47)	29, 65			
**1**	16/33 (48)	31, 66	13/30 (43)	26, 62	16/30 (53)	35, 71			
**POCTA vs. POCTAch (**McNemar’s**)**	ꭓ^2^ = 0.75, df = 1, *p*-value = 0.3865		ꭓ^2^ = 0, df = 1, *p*-value = 1		ꭓ^2^ = 0, df = 1, *p*-value = 1				
**L0/R1_POCMM**							*p*-value = 0.4502, OR = 1.58 (95% CI 0.52, 4.88)	*p*-value = 0.2034, OR = 2.10 (95% CI 0.68, 6.71)	ꭓ^2^ = 0.083333, df = 1, *p*-value = 0.7728
**0**	17/33 (52)	34, 69	12/30 (40)	23, 59	10/30 (33)	18, 53			
**1**	16/33 (48)	31, 66	18/30 (60)	41, 77	20/30 (67)	47, 82			
**L0/R1_POCMMch**							*p*-value = 0.1292, OR = 0.44 (95% CI 0.14, 1.37)	*p*-value = 0.2045, OR = 0.50 (95% CI 0.16, 1.57)	ꭓ^2^ = 0, df = 1, *p*-value = 1
**0**	10/33 (30)	16, 49	15/30 (50)	33, 67	14/30 (47)	29, 65			
**1**	23/33 (70)	51, 84	15/30 (50)	33, 67	16/30 (53)	35, 71			
**POCMM vs. POCMMch** **(**McNemar’s**)**	ꭓ^2^ = 2.4, df = 1, *p*-value = 0.1213		ꭓ^2^ = 0.30769, df = 1, *p*-value = 0.5791		ꭓ^2^ = 0.75, df = 1, *p*-value = 0.3865				
**A0/P1_BAR**									
**0**	22/33 (67)	48, 81	16/30 (53)	35, 71	19/30 (63)	44, 79	*p*-value = 0.3127, OR = 1.73 (95% CI 0.56, 5.49)	*p*-value = 0.7978, OR = 1.16 (95% CI 0.36, 3.70)	ꭓ^2^ = 0.30769, df = 1, *p*-value = 0.5791
**1**	11/33 (33)	19, 52	14/30 (47)	29, 65	11/30 (37)	21, 56			
**A0/P1_BARch**									
**0**	22/33 (67)	48, 81	17/30 (57)	38, 74	16/30 (53)	35, 71	*p*-value = 0.4472, OR = 1.52 (95% CI 0.49, 4.82)	*p*-value = 0.3127, OR = 1.73 (95% CI 0.56, 5.49)	ꭓ^2^ = 0, df = 1, *p*-value = 1
**1**	11/33 (33)	19, 52	13/30 (43)	26, 62	14/30 (47)	29, 65			
**BAR vs. BARch** **(**McNemar’s**)**	ꭓ^2^ = 0, df = 1, *p*-value = 1		ꭓ^2^ = 0, df = 1, *p*-value = 1		ꭓ^2^ = 0.36364, df = 1, *p*-value = 0.5465				
**L0/R1_TORS**							*p*-value = 0.8025, OR = 0.83 (95% CI 0.27, 2.48)	*p*-value = 0.616, OR = 0.72 (95% CI 0.24, 2.17)	ꭓ^2^ = 0, df = 1, *p*-value = 1
**0**	16/33 (48)	31, 66	16/30 (53)	35, 71	17/30 (57)	38, 74			
**1**	17/33 (52)	34, 69	14/30 (47)	29, 65	13/30 (43)	26, 62			
**L0/R1_TORSch**							*p*-value = 0.6164, OR = 1.34 (95% CI 0.44, 4.12)	*p*-value = 0.3137, OR = 1.74 (95% CI 0.58, 5.39)	ꭓ^2^ = 0, df = 1, *p*-value = 1
**0**	20/33 (61)	42, 77	14/30 (47)	29, 65	14/30 (47)	29, 65			
**1**	13/33 (39)	23, 58	16/30 (53)	35, 71	16/30 (53)	35, 71			
**TORS vs. TORSch** **(**McNemar’s**)**	ꭓ^2^ = 0.5625, df = 1, *p*-value = 0.4533		ꭓ^2^ = 0.16667, df = 1, *p*-value = 0.6831		ꭓ^2^ = 0.36364, df = 1, *p*-value = 0.5465		**TORS vs. TORSch** **(**McNemar’s**)**		

Notations: The deviation of overlapping coefficients to the left (L0) or to the right (R1), anterior (A0) or posterior (P1), during MVC and chewing (ch). n/N (%) represents the number of subjects with specific deviations (n) related to the total number of subjects in the group (N), i.e., the relative frequency (as percentage).

## Data Availability

Data supporting reported results can be found at https://github.com/arnautoleg/Mostovei-Mihai (accessed on 1 June 2022).

## References

[1] Cunha-Cruz J., Hujoel P.P., Nadanovsky P. (2007). Secular Trends in Socio-economic Disparities in Edentulism: USA, 1972–2001. J. Dent. Res..

[2] Al-Rafee M. (2020). The epidemiology of edentulism and the associated factors: A literature Review. J. Family Med. Prim Care.

[3] Tsakos G., Herrick K., Sheiham A., Watt R.G. (2010). Edentulism and Fruit and Vegetable Intake in Low-income Adults. J. Dent. Res..

[4] Lee J.S., Weyant R.J., Corby P., Kritchevsky S.B., Harris T.B., Rooks R., Rubin S.M., Newman A.B. (2004). Edentulism and nutritional status in a biracial sample of well-functioning, community-dwelling elderly: The Health, Aging, and Body Composition Study. Am. J. Clin. Nutr..

[5] Thomason J.M., Kelly S.A.M., Bendkowski A., Ellis J.S. (2012). Two implant retained overdentures––A review of the literature supporting the McGill and York consensus statements. J. Dent..

[6] Zembic A., Wismeijer D. (2014). Patient-reported outcomes of maxillary implant-supported overdentures compared with conventional dentures. Clin. Oral Impl. Res..

[7] Ülkü S.Z., Acun Kaya F., Uysal E., Gulsun B. (2017). Clinical Evaluation of Complications in Implant-Supported Dentures: A 4-Year Retrospective Study. Med. Sci. Monit..

[8] Nishi S.E., Basri R., Alam M.K. (2016). Uses of electromyography in dentistry: An overview with meta-analysis. Eur. J. Dent..

[9] Chang D., Padilla M., Hargens A., Popescu L.M., Hargens A.R., Singal P.K. (2014). Surface electromyography: Technical developments and clinical applications in sports medicine. Adaptation Biology and Medicine.

[10] Klasser G.D., Okeson J.P. (2006). The clinical usefulness of surface electromyography in the diagnosis and treatment of temporomandibular disorders. J. Am. Dent. Assoc..

[11] Marotta N., Ferrillo M., Demeco A., Drago Ferrante V., Inzitari M.T., Pellegrino R., Pino I., Russo I., de Sire A., Ammendolia A. (2022). Effects of Radial Extracorporeal Shock Wave Therapy in Reducing Pain in Patients with Temporomandibular Disorders: A Pilot Randomized Controlled Trial. Appl. Sci..

[12] Ferrario V.F., Sforza C., Colombo A., Ciusa V. (2000). An electromyographic investigation of masticatory muscles symmetry in normo-occlusion subjects. J. Oral Rehabil..

[13] Ximinis E., Tortopidis D. (2018). Electromyographic activity changes of jaw-closing muscles in patients with different occlusion schemes after fixed prosthetic restoration. Balkan J. Dent. Med..

[14] de Rossi M., Palinkas M., Lucas B., Santos C., Semprini M., Oliveira L., Regalo I., Bersani E., Migliorança R., Siéssere S. (2017). Masticatory muscle activity evaluation by electromyography in subjects with zygomatic implants. Med. Oral Patol. Oral Cir. Bucal..

[15] Elsyad M.A., Hegazy S.A.F., Hammouda N.I., Al-Tonbary G.Y., Habib A.A. (2014). Chewing efficiency and electromyographic activity of masseter muscle with three designs of implant-supported mandibular overdentures. A cross-over study. Clin. Oral Impl. Res..

[16] Al-Magaleh W.R., Abbas N.A., Amer A.A., Abdelkader A.A., Bahgat B. (2016). Biting Force and Muscle Activity in Implant-Supported Single Mandibular Overdentures Opposing Fixed Maxillary Dentition. Implant. Dent..

[17] von der Gracht I., Derks A., Haselhuhn K., Wolfart S. (2017). EMG correlations of edentulous patients with implant overdentures and fixed dental prostheses compared to conventional complete dentures and dentates: A systematic review and meta-analysis. Clin. Oral Impl. Res..

[18] Dellavia C., Francetti L., Rosati R., Corbella S., Ferrario V.F., Sforza C. (2012). Electromyographic assessment of jaw muscles in patients with All-on-Four fixed implant-supported prostheses. J. Oral Rehabil..

[19] Ferrario V.F., Tartaglia G.M., Maglione M., Simion M., Sforza C. (2004). Neuromuscular coordination of masticatory muscles in subjects with two types of implant-supported prostheses. Clin. Oral Implant. Res..

[20] Goodacre C.J., Bernal G., Rungcharassaeng K., Kan J.Y.K. (2003). Clinical complications with implants and implant prostheses. J. Prosthet. Dent..

[21] Craciunescu E., Sinescu C., Negrutiu M.L., Pop D.M., Lauer H.-C., Rominu M., Hutiu G., Bunoiu M., Duma V.-F., Antoniac I. (2016). Shear Bond Strength Tests of Zirconia Veneering Ceramics after Chipping Repair. J. Adhes. Sci. Technol..

[22] Karkazis H.C. (2002). EMG activity of the masseter muscle in implant supported overdenture wearers during chewing of hard and soft food. J. Oral Rehabil..

[23] Tanaka M., Bruno C., Jacobs R., Torisu T., Murata H. (2017). Short-term follow-up of masticatory adaptation after rehabilitation with an immediately loaded implant-supported prosthesis: A pilot assessment. Int. J. Implant Dent..

[24] Bakke M., Holm B., Gotfredsen K. (2002). Masticatory function and patient satisfaction with implant-supported mandibular overdentures: A prospective 5-year study. Int. J. Prosthodont..

[25] Saini M. (2015). Implant biomaterials: A comprehensive review. WJCC.

[26] Gartner J.L., Mushimoto K., Weber H.-P., Nishimura I. (2000). Effect of osseointegrated implants on the coordination of masticatory muscles: A pilot study. J. Prosthet. Dent..

[27] Weber H.-P., Morton D., Gallucci G.O., Roccuzzo M., Cordaro L., Grutter L. (2009). Consensus statements and recommended clinical procedures regarding loading protocols. Int. J. Oral Maxillofac. Implant..

[28] Erdelyi R.-A., Duma V.-F., Sinescu C., Dobre G.M., Bradu A., Podoleanu A. (2020). Dental Diagnosis and Treatment Assessments: Between X-rays Radiography and Optical Coherence Tomography. Materials.

[29] Erdelyi R.-A., Duma V.-F., Sinescu C., Dobre G.M., Bradu A., Podoleanu A. (2021). Optimization of X-ray Investigations in Dentistry using Optical Coherence Tomography. Sensors.

[30] Giannakopoulos N.N., Corteville F., Kappel S., Rammelsberg P., Schindler H.J., Eberhard L. (2017). Functional adaptation of the masticatory system to implant-supported mandibular overdentures. Clin. Oral Impl. Res..

[31] Uram-Tuculescu S., Cooper L., Foegeding E., Vinyard C., De Kok I., Essick G. (2015). Electromyographic Evaluation of Masticatory Muscles in Dentate Patients Versus Conventional and Implant-Supported Fixed and Removable Denture Wearers—A Preliminary Report Comparing Model Foods. Int. J. Prosthodont..

[32] Trulsson M. (2005). Sensory and motor function of teeth and dental implants: A basis for osseoperception. Clin. Exp. Pharmacol. Physiol..

[33] Ferrario V.F., Sforza C. (1996). Coordinated electromyographic activity of the human masseter and temporalis anterior muscles during mastication. Eur. J. Oral Sci..

[34] Alqutaibi A.Y., Kaddah A.F., Farouk M. (2017). Randomized study on the effect of single-implant versus two-implant retained overdentures on implant loss and muscle activity: A 12-month follow-up report. Int. J. Oral Maxillofac. Surg..

[35] Fontijn-Tekampl E., Slagter A.P., van’t Hof M.A., Geertman M.E., Kalk W. (1998). Bite Forces with Mandibular Implant-retained Overdentures. J. Dent. Res..

[36] Uçankale M., Akoğlu B., Özkan Y., Ozkan Y.K. (2012). The effect of different attachment systems with implant-retained overdentures on maximum bite force and EMG: EMG activity of different overdenture system. Gerodontology.

[37] Elsyad M.A., El-asfahani I.A., Kortam S.A., Mourad S.I. (2021). Masseter muscle activity of conventional denture, fixed prosthesis, and milled bar overdenture used for All-on-4 implant rehabilitation: A within-subject study. Clin. Implant. Dent. Relat. Res..

[38] Misch C. (2005). Dental Implant Prosthetics.

[39] Jacobs R., van Steenberghe D. (1993). Masseter muscle fatigue during sustained clenching in subjects with complete dentures, implant-supported prostheses, and natural teeth. J. Prosthet. Dent..

[40] De Rossi M., Santos C.M., Miglioranca R., Regalo S.C.H. (2014). All on Four^®^ Fixed Implant Support Rehabilitation: A Masticatory Function Study: All on Four^®^ and Masticatory Function. Clin. Implant. Dent. Relat. Res..

[41] Mostovei M., Mostovei A., Tiutiucă C., Dimofte A.R., Oleg A., Oleg S. (2022). Determination of masticatory efficiency in patients with fixed full implant-supported prostheses: Dynamic study. Rom. J. Oral Rehabil..

[42] Sinescu C., Duma V.-F., Dodenciu D., Stratul S., Manole M., Draganescu G.E. (2018). Mechanical properties of the periodontal system and of dental constructs deduced from the free response of the tooth. J. Healthc. Eng..

